# Analysis of Human Geography Space Based on Geographic Information System in the Context of Rural Revitalization

**DOI:** 10.1155/2022/6217760

**Published:** 2022-03-09

**Authors:** Ershen Zhang, Yajuan Zhou, Jiajun Qiao, Wei Wang

**Affiliations:** ^1^College of Geography and Environmental Science, Henan University, Kaifeng 475004, China; ^2^School of Resource and Environmental Sciences, Wuhan University, Wuhan 430079, China; ^3^School of Culture Industry and Tourism Management, Henan University, Kaifeng 475004, China

## Abstract

Nowadays, it is proposed “Firmly implement the rural revitalization strategy”, and rural revitalization has risen to the level of national-level strategy. Human geography is to respond to the cultural effects produced by various residents with similar living habits together. As a spatial science, GIS, with its unique spatial viewpoint and spatial thinking, reveals the spatial distribution characteristics and dynamic change laws of various things and phenomena from spatial interconnections and interactions. From the spatial objects studied by GIS, this paper puts forward thoughts on the new directions of GIS development: expanding from Earth space to cosmic space, it is necessary to build coordinate system and cosmic geospatial information system, lunar geospatial information system, etc.; extending from outdoor space to indoor space, it is necessary to develop indoor geospatial information system and expand to underwater space and underground space; from macro to micro space, we can develop sports geospatial information system for games, human geospatial information system for life and health management, etc.; facing the era of big data, we can develop theories and methods of spatial analysis of big data and contribute to the development of big data science. Thus, the way of constructing GIS based on human geospatial analysis can be built in the context of the era of rural revitalization. The purpose of the research in this paper is mainly to show the role of GIS in the construction of human geography space. Through GIS means, it can better gather some villages in human geography, have a better form of expression, and can better construct a spatial information system.

## 1. Introduction

Traditional geospatial information systems mainly study the Earth's surface system, focusing on the surface space closely related to human production and life, with geographic science and mapping science as the main disciplinary bases and the extensive use of information technology. With the application of geospatial information systems, the subdisciplines of Earth sciences such as atmospheric science [1], marine science, environmental science, and geology have also gradually applied geospatial information systems as a technical means to process and analyze complex spatial data and to make maps. In the field of medicine, geospatial information systems are widely used to discover the relationship between the environment and life and health, such as the relationship between cancer and environmental pollution and the relationship between children's congenital birth defects and the natural environmental background. In the field of criminology, geospatial information systems are used to analyze the environmental characteristics of crimes and trace the spatial and temporal clues of crimes. These applications have contributed to the development of GIS-related subfields, such as transportation geospatial information systems, urban geospatial information systems, land geospatial information systems. The deepening application of GIS in the field of Earth science has promoted the expansion of the theoretical approach of GIS from two-dimensional plane space to three-dimensional Earth space based on the geocentric coordinate system. For the future “Come to Earth” project, GIS needs to further develop various dynamic process models based on spatial analysis models, coupled with the Earth system simulator to realize the dual model system of data-driven and model-driven, and it is believed that the GIS based on human geospatial can be well constructed after this research [2].

Based on the strategy of rural revitalization, this paper constructs a spatial structure of geographic information based on human geography, adopts a combination of field research and theoretical study, analyzes the rural development problems [3], analyzes the connotation of characteristic rural villages in the context of rural revitalization, and combines practical cases. It is widely used in the fields of resources and environment, disaster and emergency response, economic and social development, health and life, rural planning and regional design, etc., and has become an indispensable and important part of today's information society. Facing the development trend of new generation IT science and technology such as mobile Internet, big data, and cloud computing, the development of geographic information systems faces new strategic opportunities and challenges. From the perspective of spatial thinking of geographic information systems, this paper considers the expanding fields of geospatial information systems, ponders the key scientific and technical issues, and builds a world of geospatial information systems that is omnipresent. The paper proposes targeted rural planning strategies to better promote the construction of distinctive rural villages and achieve the goal of rural revitalization [4].

As a typical regional economic prosperity area, rural village planning in Area A started earlier and has made more achievements and experiences. Under the policy guidance and promotion of rural construction actions, exploring the rural planning strategy of characteristic field villages in the context of rural revitalization is an important way to solve the current problems of rural villages and guide the construction of characteristic field villages and realize rural revitalization and has a strong typical significance and demonstration role.

## 2. Related Work

About the content of rural revitalization research, one is the different opinions on the connotation of village rural planning. The literature considers village rural planning as a “village regulation”, and the comprehensive development, construction, and governance of villages play a guiding and regulating role; the literature considers that village rural planning has the characteristics and role of rural governance. The second is the exploration of village rural planning methods, one is the study of village rural planning standards, the literature puts forward the corresponding rural planning control and guidance on rural land classification, public facilities configuration [5], etc. One is to explore innovative rural planning methods for rural planning characteristics, literature exploration “all-round,”and the other is to explore innovative rural planning methods for rural planning characteristics. In terms of research perspectives, one is on urban-rural relationship perspective, combining with current hot topics, such as new rural construction, new urbanization, urban-rural integration, discussing rural-rural planning methods, rural planning system, and rural planning design strategies. The literature explores rural-rural planning values, cognitive research, and technical methods under the perspective of urban-rural relationship; the second is on cultural perspective, exploring the essential characteristics of rural villages, such as the literature proposes that rural modernity and traditionalism should be realized in rural planning, guiding vernacular spatial rural planning and architectural construction. For rural revitalization, rural countryside planning research started earlier and developed more maturely. In terms of rural space, the research focuses on land use, road systems, farm buildings, and other aspects, explores the multifunctional use of land and landscape in rural countryside planning, integrates different land uses and rural landscapes, focuses on rural space environmental restoration, and believes that rural countryside planning should consider the balance relationship between the ecological environment and macro-space. The literature studies urban-rural boundaries reduce the drawbacks of urban sprawl and safeguard rural green space for sustainable development [6].

In terms of rural character, the rural character is gradually fading due to urban influence, and the preservation of rural character has also become an important rural planning objective in many rural areas, through public perception, focusing on the selection of appropriate rural development strategies to protect rural character based on the understanding of farmers' needs and attitudes towards the rural character. The literature argues that rural character should be understood based on visual perception, and the reasons for its formation should be analyzed. In terms of rural social governance, the role of multiactor governance is recognized to understand the strengths and weaknesses of rural planning and construction projects by investigating the perceptions of actors involved in rural areas, and participatory rural planning methods are used to involve local villagers in the development and implementation of rural planning to promote sustainable efficiency in agricultural modernization and sustainable rural development. Literature, rural village planning management should pay attention to the influence of the agricultural sector and prepare and implement rural planning with farmers as the guide; the literature studies the role of multiactor governance in agricultural modernization and sustainable rural development.

For geospatial information analysis systems, since the industrial revolution, human's ability and intensity to develop valuable natural resources have been increasing, bringing huge economic benefits to the real society, while the global ecological and environmental problems have become more and more prominent. With the flourishing of computer technology, GIS, as a new technology with strong professionalism, began to be concerned by many ecological environmentalists. In the 1960s, some schools and institutions in foreign countries began to do exploratory research on the application of GIS technology to environmental resources. The developed countries have applied GIS technology to the environmental management field earlier and have been in the leading position in the basin water environment regulation technology for a long time. More representative countries are the United States, the Netherlands, Canada, Australia, France, Germany, and the former Soviet Union, and they have achieved certain success in the use of GIS technology to address the field of environmental resources.

## 3. Exploration of Rural Revitalization and Geospatial Information System Construction

**Figure 1:**
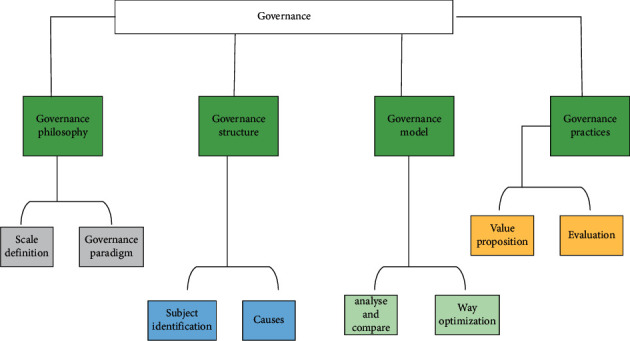
Village governance model.

Throughout the global research results, scholars from different disciplines have conducted in-depth research and practice on rural village planning and construction from multiple perspectives and levels, and the breadth of research and depth of practice have been continuously improved. In terms of rural planning theory, experts and scholars at home and abroad have conducted fruitful research on rural planning, more comprehensive analysis of rural planning concepts, and discussions on rural planning strategies and rural planning management, adding a large number of research results [7], which have a significant role in promoting rural planning construction and rural planning research. Rural planning research gradually gets rid of the single material space rural planning theory, deepens rural planning basic research, analyzes the connotation of rural planning under different perspectives, and explores rural planning methods. And foreign rural village planning pays more attention to the characterization and quality of rural village planning and construction, emphasizes the systematic nature of rural planning design and rural planning implementation, and emphasizes top-down macro-control and bottom-up public participation, which has a greater reference and reference role for rural village planning and construction. For the governance of villages, its model is shown in Figure 1.

### 3.1. Selection of Characteristic Elements of Rural Revitalization

**Figure 2:**
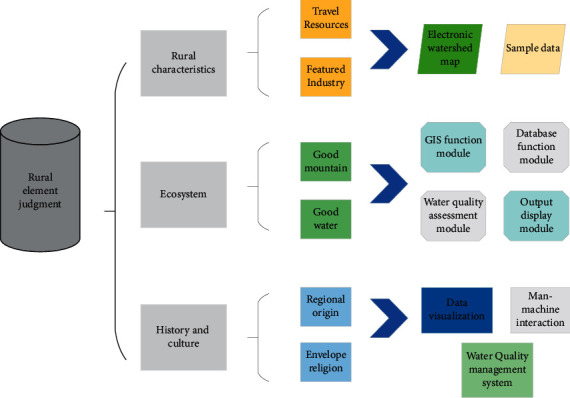
Selected elements of rural revitalization characteristics.

According to the requirements of the characteristic rural village planning and construction objectives, the characteristic elements of the countryside should be sorted out, the laws of rural development in the specific regional environment should be explored, and the uniqueness of the countryside should be explored [8], to serve as the basis for rural revitalization. The countryside in Zone A has strong regional characteristics, with its special geographical location, superior industrial base, unique ecological environment, and profound cultural heritage, which impose requirements on rural economic, ecological, cultural, political, social development, and other aspects requirements. The industrial characteristics, ecological characteristics, and cultural characteristics of the countryside are analyzed. The characteristic elements of rural revitalization are selected as shown in Figure 2.

The focus is on the improvement of domestic garbage, domestic sewage, industrial pollution sources, agricultural waste, river ditches, and ponds, to achieve daily cleaning and timely, and establish a long-term mechanism, efficient use of resources and environment, improve the level of rural environment greening, effectively improve the quality of the village ecological environment, focus on improving public facilities supporting, greening, and beautification, drinking water security, road access, building style and characterization, village environmental management level, to protect the villagers' daily life convenience. Through village improvement and rural planning, the appearance of villages in District A has changed greatly, the ecological environment has improved greatly, the facilities are more complete, the daily life is increasingly rich, the quality of villagers' production and life has been improved, the ecological and social benefits of villages have played or are playing a positive role, and District A has fully started and completed the village environmental improvement work within one year in Jiangsu Province and has accumulated more than 10,000 villages for improvement. More than 10,000 villages have been improved [9].

### 3.2. Rural Development Pathways

**Figure 3:**
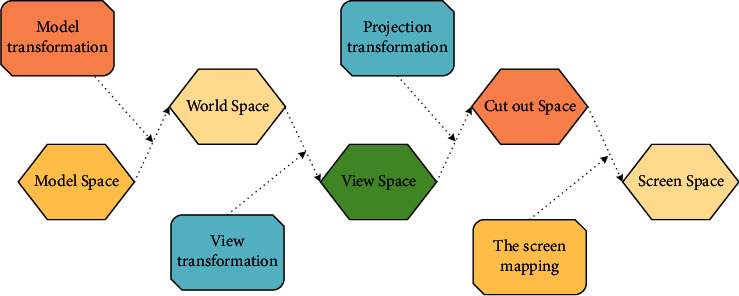
Spatial coordinate conversion process.

**Figure 4:**
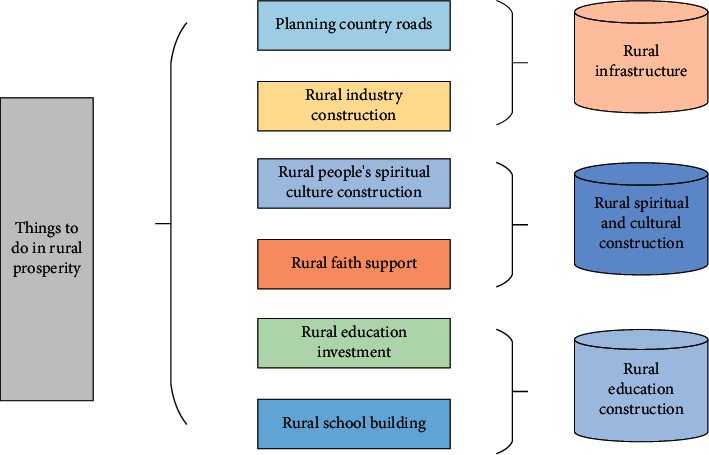
Logic of rural governance.

(1) To solve the problem of the industry being special but not strong. District A is rich in industrial characteristics and resources, with a large potential for industrial development, and has the foundation for the development of a special rural village, but the output value brought by agriculture is low, and the overall special but not strong, and is in urgent need of transformation and upgrading. From the viewpoint of industrial integration, the association between one, two, and three industries in the villages of District A is not strong and shallow and has not yet reached the requirements of rural revitalization. In terms of primary industry, some villages have special agriculture, but the output is generally not high, and the income of the agricultural economy is low [10], resulting in the phenomenon of weakening agricultural production and desolation of land; in terms of secondary industry, it mainly undertakes low-end industries from the city and obtains more collective economic income through plant leasing and land leasing, but the consequent environmental pollution and rising labor price make the development of villages weak; in terms of the status quo of tertiary industry, the development content is single, lack of service support, mainly sightseeing type rural tourism, mostly farm caravans, lack of experience type activities set. How to express the features in 3D scene through the two-dimensional screenand WebGL rendering pipeline? Firstly, the vertex picked up the model spatial coordinates; after that spatial coordinate transformations are multiplies to get the pixel point coordinates, and finally, it is projected onto the two-dimensional screen [11]. The whole transformation process is shown in Figure 3.
(2) To solve the problem of unsatisfactory ecological habitat, in the process of rural development. Some villages in District A focus on collective village economic income and even implement the policy of “development first and governance later”, which has caused greater damage to the rural ecological environment. In recent years, although the environmental protection of villages in District A has been strengthened, most of the villages are mainly improved by planting trees and painting walls, while the maintenance of rural habitat is neglected, resulting in damage to the spatial texture and some functions lagging. In terms of damaged spatial texture, rural rivers are not sufficiently maintained and are blocked and polluted by a large amount of production and living waste [12], the spatial texture adjacent to traditional rivers and streets is broken, and the quality of villagers' living environment is reduced; the ecological space such as farmland and woodland is encroached upon by construction land, residential and factory land is mixed, the continuity and integrity of spatial texture are damaged, and the ecological pattern is seriously affected. In terms of functional lag and residential function, the traditional residential space does not match modern life, and the urbanized centralized public space rural planning and design do not meet the villagers' habits of interaction and living nearby; in terms of service function, the villages lack production facilities such as primary production of agricultural products and deep processing space, and living facilities such as elderly facilities and related support, and the development of service support cannot keep up with the speed of rural development; in terms of transportation function, some agricultural production roads are missing, unable to meet the basic user needs of villagers, affecting the stability of villagers' lives and the convenience of production, and seriously reducing the quality of life. The essential principle is shown in the formula [13].
  (1) $$\begin{array}{c} T=\frac{\delta y}{\delta x}.\frac{\Delta y}{\Delta x}.\frac{\partial^{2}\Omega }{\partial u\;\partial v}. \end{array}$$
As the key formula of the article, the importance of the first formula in the article is self-evident. The degree of application of GIS to the human geographic space can directly reflect the level of efficiency.
(3) To solve the problem of poor cultural heritage. The rural villages in District A are rich in cultural heritage and distinctive and are the emotional support of villagers' “nostalgia”. However, the research found that in the process of rural development in District A, there is a lack of protection and restoration of material cultural space, and the transmission of intangible culture is difficult. On the one hand, the countryside is rigidly applying the village construction guidelines, blindly imitating the city, neglecting the protection of historical buildings and other material culture, and lacking the excavation and extraction of the countryside's cultural characteristics, resulting in the alienation of the countryside's architectural appearance, the disappearance of traditional vernacular architectural features of whitewashed walls and tiles, and a reduced sense of belonging to the village; on the other hand, part of the vernacular culture is not inherited, traditional rituals are crumbling, traditional public spaces are gradually declining, and the intangible cultural heritage lacks attractiveness and bearing. Material culture heritage lacks attraction and bearing space. The loss of material culture protection and the endangerment of intangible cultural heritage make villagers gradually lose their identity and village life memory, making it difficult to meet the multilevel and multifaceted cultural needs of the peasants, which is not conducive to building a spiritual home in the villages of District A. Its essential principle is shown in the following formula:
  (2) $$\begin{array}{c} A=\frac{1}{n}\sum_{i=1}^{n}{\left(X_{i}-\bar{X}\right)}^{2}+\frac{x-\mu }{\sigma }. \end{array}$$
As the key formula of the article, the second formula in the article indicates the availability of GIS, which can directly reflect the level of efficiency.
(4) To solve the problem of inadequate rural governance: The rural village planning and construction movement in District A are in full swing but still face the problem of inadequate rural governance due to the scattered efforts of higher-level departments, such as beautiful rural village planning managed by the Agricultural Office, statutory rural village planning managed by the Rural Planning Bureau, and rural village planning with special characteristics managed by the Housing and Urban Development Bureau, as well as the lack of awareness of villager participation. Provincial, municipal, district, and town government departments at all levels have been exploring solutions to the “three rural issues” and have gained a lot of experience, but due to scattered inputs and separate management, some of the work is duplicated, and it is difficult to focus on the advantages of various departments, which is inefficient and ineffective. Due to the influence of the work of Jiangsu Province around the “three rural areas”, the “three rural areas” work in city A is not focused enough, and the government can hardly play the most effective role in rural governance [14]. The governance logic is shown in Figure 4.

## 4. Construction of a Human Geospatial System Analysis of Intelligent Distribution Results

**Figure 5:**
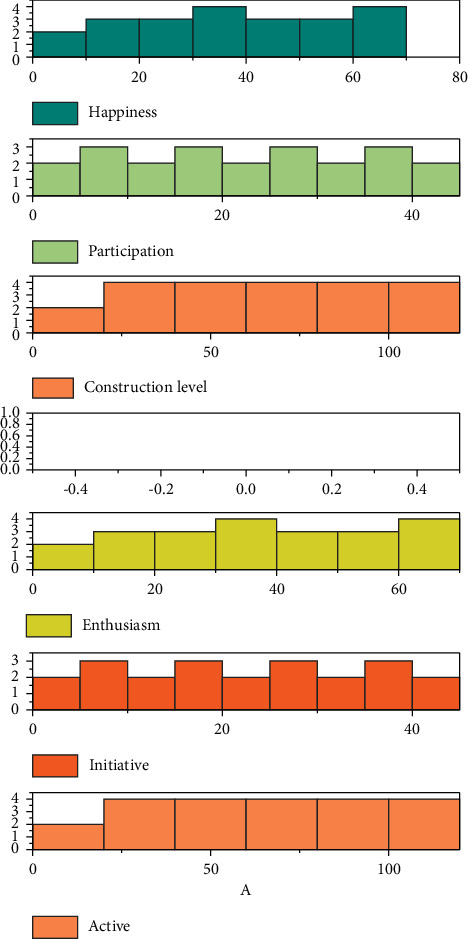
Various emotions of the villagers.

It was an important international trend at that time to standardize water quality data by using the powerful capability of GIS technology to display, process, and analyze watershed spatial information. The vigorous development of computer information technology, especially the emergence of 3S (GIS, RS, GPS) technology, Internet technology, and expert system, has provided new vitality to the water environment and the water environment supervision information system. Foreign countries have carried out a lot of exploratory activities in using 3S technology to build a water environment supervision information system [15].  Figure 5 shows the various emotions of the villagers.

### 4.1. Human Geospatial Information System Component Composition

ArcGIS Engine (ArcEngine) is a new generation product launched by ESRI after ArcGIS version 9.0. ArcEngine is a set of component repositories with major GIS functions, and secondary developers can topology new functions from the original software. ArcObjects is the core library of ArcGIS software, with over 3,000 controls available for secondary developers. The ArcEngine component library is the core library of ArcGIS software, with more than 3000 controls available for secondary developers to use, allowing them to quickly develop GIS projects. The ArcEngine component library can not only develop and implement basic GIS functions but also integrate some professional technical models, which can achieve the following functions: map zoom in, zoom out, roaming, layer deletion overlay, etc.; store and read MDX files; data display, symbolization, thematic map production and other map representation; description text, graphic elements; coordinate conversion; mouse slide over to display elements or by point-line surface recognition; mouse selection and highlighting of elements; displaying only filtered elements; drawing geometric shapes; free rotation of the map and reversal of the map; development of personalization tools; pop-up color picker; display of layer properties window; styling of page layouts and creation of thematic maps; tracking of GPS property elements; gradient colors of the map; adding field property information; statistical analysis of data; viewing whether the map is in statistical analysis; viewing whether the map is in editing status [16].

### 4.2. Human Geospatial Information System Algorithmic Framework

The most important feature of rural country planning information is that it has a huge amount of data, a large variety of logical structures, and the same.

When there are temporal and spatial characteristics, the previous data storage methods are difficult to supervise quickly and reasonably, but the storage and processing of these massive data through database technology as well as computer technology can make rural management [17].

It is more brief and efficient. Its advantages are specifically.

#### 4.2.1. Structured Data

**Figure 6:**
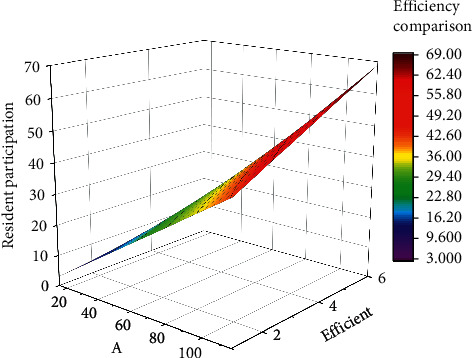
Efficiency comparison chart.

The fundamental difference between the database and the earlier Windows file system is that the database can be used to reduce the redundancy of data in the system as well as to coordinate the uniform scheduling between data by splitting and then reassembling data with complex structure and finally expressing the data in such a way that not only the data itself can be described [18] but also the relationships that exist between the data as shown in Figure 6. Theefficiency comparison diagram of the village construction process shows whether the construction of the human geospatial information system is carried out.

#### 4.2.2. Data Sharing

Data shareability is mainly manifested by the fact that data can be used by users together at the same time [19]; there is compatibility and inconsistency between data, and the database storage space is large and easy to fill to meet user needs. The essential principle is shown in the following formula:

(3) $$\begin{array}{c} F=\frac{1}{n}\sum_{i=1}^{n}X_{i}.\left(\frac{x-\mu }{\sigma }\right). \end{array}$$

#### 4.2.3. Independence of Data

The independence of the application and the data structure from each other is one of the most important goals of a database system. It makes the data independent of the application, and it removes the data definition statements from the project program, and because the access to the data is handled by the DBMS, it greatly facilitates the preparation of the application and greatly reduces the error rate and maintenance costs of the application.

#### 4.2.4. Centralized Data Control

The data are managed and controlled by the DBMS, with its uniform data types, data logic structure, so that the datahave good unity, security, integrity, and other functions.

### 4.3. Human Geospatial Information System Data Framework

There are two types of data designed for the system, one is spatial data and the other is nonspatial data (i.e., attribute data) [20]. The geospatial data are primarily a representation of geospatial information about the watershed in Area A, such as the perimeter of Area A.

The logical structure of the spatial data content is its relationship with the GIS, such as roads, river systems, monitoring stations.

The system is used to establish a link, build a scientific and reasonable data structure, and makeeffectively use the GIS function. Property data refer to various water quality measurement values, water quality standard values, and statistical analysis results of the records related to the watershed. The implementation of a database management information system is based on various types of data, so the data concept model is the core part of building a database system. With the database management system, the user can deal with data in the abstract sense without regard to the layout and physical location of these data in the computer. The model of the water environment information management system in area A is the awareness and abstraction of the water environment in the actual area A. The design of the water environment database in area A has several data tables on the subject, and the names and contents of the data tables are shown in Table 1.

## 5. Experimental Procedure and Analysis

### 5.1. Experimental Content

To discuss whether constructing a spatial information system of human geography is helpful for rural revitalization, this paper makes an experiment based on it. The spatial pattern is the externalized form of spatial connotation [21], and the pattern can reveal the structure and mechanism of things as well as provide the basis and motivation for the new development of things. On this basis, the spatial network pattern of rural life is a typical spatial schema shaped by human/nonhuman subjects in a certain region based on the network structure through the network mechanism. It contains the three-dimensional connotation of livelihood protection, quality improvement, and harmony between humans and land.

The system used for experiments is an application system based on ArcEngine components, and the design idea is based on the actual needs of water and environment management in Area A. Practicality, reliability and rationality are the leading ideas, so the development process of the system follows the following principles.

1. Practicality: The design and construction of the system need to be based on the actual work needs, to meet the requirements of the relevant management departments for the storage, query, and statistical analysis functions of monitoring data, and ultimately to provide scientific-technical support for the rational rural planning and evaluation of the lake. At the same time, the hierarchical structure of the system should be clear, functional use and simple and easy to understand user interface.
2. Normality: The coding of data is standardized, and the form of information storage is standardized. The design of the structure of the spatial geographic database and the coding form of the attribute data follow the relevant standards set by the State [22].
3. Openness: The system is designed following the principle of openness, capable of responding to various software and hardware devices and network information systems, and its software system and drivers support secondary development. The systems adopt international standard data interfaces, and the system should also have the ability to share data and correlate data with other information management systems. At the same time, the development environment of the system selects the mainstream platform, which facilitates the upgrade of the system in the future. The essential principle is shown in the formula.
4. Economy: It is undesirable to ignore the development cost by pursuing the technical improvement only while ensuring that the system can achieve all the objectives set and that the development and application of the system can be done at the lowest cost. The system chooses the most common Windows 8 as the system running environment, to meet the principle of economy of the system.
5. Security: The end-users of the system have different operation rights according to their work requirements, and the three layers of security assurance of the software system, database management system, and application model should be fully satisfied to meet the security of the core data.

**Figure 7:**
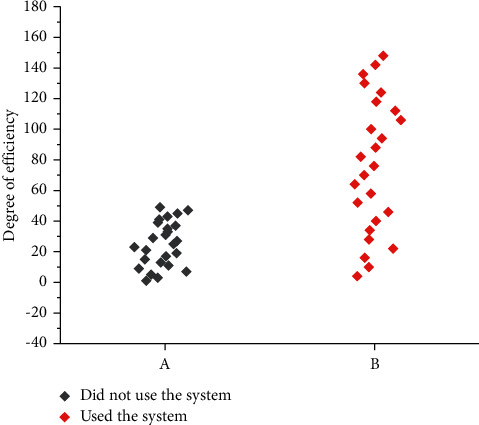
Efficiency comparison chart.

The efficiency of managing remediation in villages with or without human geographic-based geospatial information systems is shown in Figure 7.

**Figure 8:**
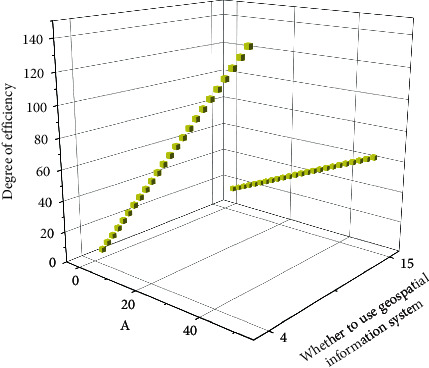
Efficiency comparison chart.

In this paper, through the daylighting analysis under the real 3D scene of the experimental system, simulating the influence of sunlight in different periods and different periods on the mutual situation between the new urban buildings and the surrounding buildings, avoiding certain floors by the shading situation cannot get a reasonable and effective sunlight duration, can provide technical reference for the design of the height and volume of new rural planning buildings and so on. The framework analyzes the implementation of sunlight and can use its sunlight characteristics to produce special effects to produce light effects on a certain area of the building at a certain time of the day. The daylight analysis can intuitively and quickly grasp the impact of light on buildings at a certain time, understand whether the layout of reconstructed buildings and surrounding buildings in urban and rural planning is reasonable, provide technical support for the decision-making of rural planning management departments, and improve the efficiency of relevant departments. In the spatial system construction and no system construction of rural countryside, planning has a different efficiency. The different efficiencies are shown in Figure 8.

## 6. Experimental Analysis

Rural living space governance is a process in which multiple actors deal with the complex supply and demand of living resources in rural territories. Governance subjects, governance objects, governance units, and governance values/goals form a complete governance framework, and the four together determine the positions and functions of different governance structures in the governance cluster. Its basic operation process is to establish a coalition of governance actors and rural governance rules in response to the governance situations generated by specific rural living space and to exert influence on the network structure of rural living space by integrating and configuring the supply and demand of key factors such as population, land, and industry. Among them, governance tools, as intermediaries that facilitate the connection and interaction between governance actors and governance objects in the process of rural living space network governance, are the core components of coordinating regional living elements, optimizing living space structure, and enhancing living space functions. The composition of governance tools of rural living space networks mainly includes spatial cohesion policy, spatial network-type planning, and spatial informal contract. Based on the combination of theory, problem, and practice, the purpose of spatial cohesion policy implementation is to coordinate the focus of government spatial policy on living functions, grasp the value of living space governance and the goal of living civilization construction, and maintain the function of rural living space network; spatial network-type planning, on the other hand, through improving the organization of rural living resources flow, promoting rural industrial development, driving the improvement of residents' income level and rural social-spatial network planning stimulate the structural organization of rural living space network by improving the organization of rural living resources flow, promoting the development of rural industries, and driving up the income level of residents and the operation of rural social capital; spatial informal contract taps into the spatial guidance and regulation of the behavior, consciousness, and social relations of rural subjects, absorbing all the elements dedicated to maximizing the marginal utility of living space. The combination of the three forms the equilibrium of supply and demand, network optimization, and development vitality of the spatial structure of rural life. For the construction of a spatial information system based on human geography, it must be better to realize the rural revitalization strategy.

## 7. Conclusion

The logical backbone of the article is “rural human-land relations, rural spatial network, and rural spatial system”. This paper presents the problems of rural human-land life relations from the perspective of rural human-land relations, establishes a theoretical medium based on rural life spatial network and portrays its structure and changes, then describes the life attributes and regulation paths of the regional rural spatial system, and then constitutes a spatial information system based on human geography. The spatial network of rural life is a spatial network of rural human-land relations constituted by the rural settlements of different scales as nodes, the multiflow life paths as linking paths, and the natural and social conditions of rural areas at a certain scale as the living background. Cutting into rural spatial systems with a relational perspective, the study of rural living space networks is of great significance to the enrichment of the spatial structure theory of rural geography.

This paper proposes the research content of this paper, based on a humanistic geospatial information system, combining tilt photogrammetry technology and 3D WebGIS technology, conducting in-depth research on key technologies such as 3D data loading visualization rendering and spatial analysis according to the actual needs, developing the experimental system of tri-realistic map information for an urban planning management application, and the design and implementation of the overall technical architecture and application functions. Multisource data integration display application, so that the system in urban planning management applications can achieve the display of rich information, data standardization and standardization, convenient sharing and exchange, update and maintenance of the effect timely.By using the auxiliary tools of the system in three-dimensional analysis, for urban planning and construction of reasonable management, it provides strong technical support forthe rational management and development of urban planning and construction.At the same time, the system is also used to develop new functions, so that the three-dimensional real-view map information system better and more widely serve the urban planning industry, promote the development of urban information, harmonious and sustainable development, and lay the foundation for the construction of the wisdom of the countryside.

## Figures and Tables

**Table 1:** Contents of the database data table.

Data table name	Data sheet content
User information form	Account password
Geographic information table	Administrative boundary
Rural topographic observation table	Remote sensing satellite influence map
Rural data statistical analysis table	Rural evaluation data

## Data Availability

The data used to support the findings of this study are available from the corresponding author upon request.
